# Supplementary material for: Content-rich biological network constructed by mining PubMed abstracts
Source: BMC Bioinformatics. 2004 Oct 8;5:147. doi: 10.1186/1471-2105-5-147 (PMC528731; doi:10.1186/1471-2105-5-147)
Supplement: Additional File 2 — The original results of the above study (non-essential files are deleted to keep the file size under the limit set by BMC bioinformatics). [file 1471-2105-5-147-S2.bz2 › chilibotAdditionalFile2/dip40/405ID10681548E10065/html/IL6_OSM.html]

 


 **IL6** and **OSM** 
  
Found 446 abstracts in PubMed, retrieved 40.  
 

 What does Google say? 
 PDF only 
| .edu only 

---

**Interactive relationship** (e.g. stimulation, inhibition, etc)

**Neutral relationship**- Oncostatin M  [ **OSM** ]   **OSM**  is an IL 6  [ **IL6** ]  type cytokine that regulates the growth of prostate cancer cells in a paracrine fashion.  Ref: 12114440 Clin Cancer Res, 2002
- Oncostatin M  [ **OSM** ]  belongs to the interleukin 6  [ **IL6** ]  family of cytokines and acts as a multifunctional cytokine during murine embryogenesis and in inflammatory reactions.  Ref: 12814362 Eur J Neurosci, 2003
- IL 6  [ **IL6** ] , leukemia inhibitory factor LIF, and oncostatin M  [ **OSM** ]   **OSM**  are IL 6  [ **IL6** ]  type cytokines that stimulate osteoclast formation and function.  Ref: 12218157 J Immunol, 2002
- Oncostatin M  [ **OSM** ]   **OSM**  is a multifucntinal cytokine that belongs to the IL 6  [ **IL6** ]  family.  Ref: 12855584 Blood< MedlineTA>Blood, 2002
- CONCLUSIONS  **OSM**  is a member of the IL 6  [ **IL6** ]  family of cytokines, which causes ligand independent activation of the AR without altering receptor expression.  Ref: 12114440 Clin Cancer Res, 2002
- Oncostatin M  [ **OSM** ]   **OSM**  is a multifunctional cytokine that belongs to the Interleukin IL 6  [ **IL6** ]  subfamily.  Ref: 12811586 Rev Physiol Biochem Pharmacol< MedlineTA>Rev Physiol Biochem Pharmacol, 2002
- Oncostatin M  [ **OSM** ]   **OSM**  is a cytokine of the interleukin 6  [ **IL6** ]  IL 6  [ **IL6** ]  family secreted by activated monocytes, and is expressed in atherosclerotic plaque.  Ref: 12138373 Blood Coagul Fibrinolysis, 2002
- Oncostatin M  [ **OSM** ]   **OSM**  is a member of the interleukin 6  [ **IL6** ]  cytokine family, which is involved in definitive hematopoiesis, the development of liver, and local inflammation.  Ref: 12831858 Neuroscience, 2003
- Compared to other members in the IL 6  [ **IL6** ]  subfamily cytokines, M17 s cysteine positions and gene organisation are similar to those of oncostatin M  [ **OSM** ]  and LIF, although amino acid identities are only 15 17%.  Ref: 12543126 Dev Comp Immunol, 2003
- CONCLUSION IL 1, but not IL 6  [ **IL6** ] , TNFalpha, or iNOS, plays an important role in joint disease induced by intraarticular gene transfer of  **OSM**  in mice.  Ref: 12794844 Arthritis Rheum, 2003
- In addition, oncostatin M  [ **OSM** ]   **OSM** , an interleukin 6  [ **IL6** ]  family cytokine, down regulated the expression of cyclin D1 and D2 in a primary culture of fetal hepatocytes in which  **OSM**  induces hepatic differentiation.  Ref: 12147685 J Biol Chem, 2002
- METHODS An adenoviral vector expressing murine  **OSM**  was injected into the joints of naive wild type mice and mice deficient for interleukin 1 IL 1, IL 6  [ **IL6** ] , tumor necrosis factor alpha TNFalpha, or inducible nitric oxide synthase iNOS.  Ref: 12794844 Arthritis Rheum, 2003
- IL 6  [ **IL6** ]  type cytokines exert their action via the signal transducers gp130, LIF receptor and  **OSM**  receptor leading to the activation of the JAK STAT and MAPK cascades.This review focuses on recent progress in the understanding of the molecular mechanisms of IL 6  [ **IL6** ]  type cytokine signal transduction.  Ref: 12773095 Biochem J< MedlineTA>Biochem J, 2003
- Members of the interleukin 6  [ **IL6** ]  family of cytokines include leukaemia inhibitory factor LIF, interleukin 6  [ **IL6** ] , interleukin 11, cardiotrophin, ciliary neurotropic growth factor, oncostatin M  [ **OSM** ]  and the recently discovered cardiotropin like cytokine NNT 1.  Ref: 12385838 J Reprod Immunol< MedlineTA>J Reprod Immunol, 2003
- We conclude that a AZA inhibits the release of  **OSM** , IL 6  [ **IL6** ] , and IL 11 exclusively in RA diseased MNC, b Patient s MNC release subnormal amounts of LIF,  **OSM** , and IL 11, and c RA derived monocytes probably down regulate  **OSM**  release by phytohemagglutinin activated MNC.  Ref: 12543079 Cytokine, 2002
- Here we show that Cdk9 interacts with gp130, the receptor of the Interleukin 6  [ **IL6** ]  IL 6  [ **IL6** ]  family of cytokines, which includes Leukemia Inhibitory Factor LIF, Oncostatin M  [ **OSM** ]   **OSM** , Ciliary Neurotrophic Factor CNTF, Interleukin 11 IL 11 and Cardiotrophin CT 1.  Ref: 12386808 Oncogene, 2002
- Stimulation of human peritoneal mesothelial cells with  **OSM**  induced phosphorylation of gp130 and  **OSM**  receptor beta, which was accompanied by activation of STAT3 and secretion of CC chemokine ligand 2 monocyte chemoattractant protein 1 and IL 6  [ **IL6** ] .  Ref: 12391243 J Immunol, 2002

**Non-interactive relationship** (e.g. studied together, co-existance, homology, etc.)

- We detected LIF, IL 11, sgp130 and a large amount of IL 6  [ **IL6** ] , but not  **OSM** , CT 1, CNTF nor IL 6R in these culture supernatants.  Ref: 12126642 Cytokine, 2002
- The related members of the interleukin 6  [ **IL6** ]  IL 6  [ **IL6** ]  family of cytokines, leukemia inhibitory factor LIF, oncostatin M  [ **OSM** ]   **OSM**  and IL 6  [ **IL6** ]  are inflammatory mediators that control differentiated cell functions as well as proliferation.  Ref: 12214267 Oncogene, 2002
- Oncostatin M  [ **OSM** ]   **OSM** , a cytokine of the interleukin 6  [ **IL6** ]  family, is expressed in rheumatoid arthritis, multiple sclerosis, multiple myeloma, and other inflammatory and neoplastic conditions.  Ref: 12730964 Glia, 2003
- Studies with cultured cells and mouse models of disease have ascribed proinflammatory and anti inflammatory functions to oncostatin M  [ **OSM** ]   **OSM**  and the related cytokine, interleukin 6  [ **IL6** ]  IL 6  [ **IL6** ] .  Ref: 12856331 J Interferon Cytokine Res, 2003
- **OSM**  but not LIF, IL 6  [ **IL6** ] , or CT 1 also induced eotaxin release by mouse lung fibroblast cultures derived from C57BL 6 mice.  Ref: 12496442 J Immunol, 2003
- IL 6  [ **IL6** ] , leukemia inhibitory factor, and oncostatin M  [ **OSM** ]  stimulate bone resorption and regulate the expression of receptor activator of NF kappa B ligand, osteoprotegerin, and receptor activator of NF kappa B in mouse calvariae.  Ref: 12218157 J Immunol, 2002
- Transdifferentiation was enhanced by 5 azacytidine treatment, and by HSLF,  **OSM**  and HGF  [ **IL6** ] .  Ref: 12821039 J Hepatol, 2003
- LIF acts on responding cells by binding to a heterodimeric membrane receptor composed of a low affinity LIF specific receptor and the gp130 receptor chain also used as the receptor for interleukin 6  [ **IL6** ] , oncostatin M  [ **OSM** ] , cardiotrophin 1, and ciliary neurotrophic factor.  Ref: 12529546 Stem Cells, 2003
- Overexpression of cyclin K and inhibition of STAT3 prevents the growth suppressive effect imposed by the interleukin 6  [ **IL6** ]  type cytokine, oncostatin M  [ **OSM** ] .  Ref: 12531804 Blood, 2003
- Leukemia inhibitory factor LIF, oncostatin M  [ **OSM** ]   **OSM** , interleukin IL 6  [ **IL6** ] , and IL 11 regulate hemopoiesis and LIF,  **OSM** , and IL 6  [ **IL6** ]  also inhibit the proliferation of myeloid leukemic cell lines through the signal transducing subunit gp130.  Ref: 12543079 Cytokine, 2002
- after IL 6  [ **IL6** ]  and  **OSM**  stimulation, gp130 dependent pathways STAT3, APR gene expression were completely blocked in the liver of these animals.  Ref: 12891556 Gastroenterology, 2003
- Here, we examined the effect of systemic administration of adenoviral Ad vectors encoding either murine  **OSM**  AdMuOSM or murine IL 6  [ **IL6** ]  AdMuIL 6 in a mouse model of colitis.  Ref: 12856331 J Interferon Cytokine Res, 2003
- Cell signaling studies showed that murine  **OSM** , LIF, IL 6  [ **IL6** ] , and CT 1 stimulated the tyrosine phosphorylation of STAT 3, suggesting STAT 3 activation is not sufficient for eotaxin induction in NIH 3T3 cells.  Ref: 12496442 J Immunol, 2003
- These include preproglucagon, APOJ, cadherin2, phosphoglucoisomerase, oncostatin M  [ **OSM** ] , PAX6, HGF  [ **IL6** ] , and cytokeratin 18.  Ref: 12606813 Mol Med, 2002
- We studied the connection between the secretion of TNF alpha, IL 6  [ **IL6** ] ,  **OSM**  and NO by hamster peritoneal macrophages animals bearing two forms of hamster transplantable melanomas of common origin but differing in growth rates, levels of differentiation and immunogenicity and the cytotoxic effect of these macrophages on the cells of those two melanoma lines.  Ref: 12659693 Int J Immunopathol Pharmacol< MedlineTA>Int J Immunopathol Pharmacol, 2002
- Effect of azacytidine in the release of leukemia inhibitory factor, oncostatin m  [ **OSM** ] , interleukin IL 6  [ **IL6** ] , and IL 11 by mononuclear cells of patients with refractory anemia.  Ref: 12543079 Cytokine, 2002
- We show here that hepatocyte growth factor HGF  [ **IL6** ]  induced early transition of albumin ALB negative stem cells to ALB positive hepatic precursors resembling hepatoblasts and then oncostatin M  [ **OSM** ]   **OSM**  promoted their differentiation to tryptophan dioxygenase TO positive mature hepatocytes.  Ref: 12702664 Development< MedlineTA>Development, 2002
- Adult mice were challenged with IL 6  [ **IL6** ] , oncostatin M  [ **OSM** ]   **OSM** , and LPS.  Ref: 12891556 Gastroenterology, 2003
- AZA down regulated  **OSM** , IL 6  [ **IL6** ] , and IL 11 release by MNC of patients but not by MNC from healthy individuals.  Ref: 12543079 Cytokine, 2002
- Murine oncostatin M  [ **OSM** ]   **OSM** , a member of the interleukin 6  [ **IL6** ]  IL 6  [ **IL6** ]  related cytokine subfamily, stimulates definitive hematopoiesis and liver development.  Ref: 12412016 Dev Dyn, 2002
- The aim of this study was to analyse the capacity of myocytes and fibroblasts isolated from human atrium to secrete IL 6  [ **IL6** ] , leukaemia inhibitory factor LIF, cardiotrophin 1 CT 1, IL 11, oncostatin M  [ **OSM** ]   **OSM** , ciliary neurotrophic factor CNTF and the soluble receptor subunits sIL 6R and sgp130 during primary culture.  Ref: 12126642 Cytokine, 2002
- Oncostatin M  [ **OSM** ]   **OSM** , an interleukin 6  [ **IL6** ]  type cytokine, induces sustained up regulation of CCAAT enhancer binding protein C EBP delta mRNA and protein in nonneoplastic HC11 mouse mammary epithelial cells.  Ref: 12479220 Mol Cancer Ther, 2002
- In contrast to AR activation by IL 6  [ **IL6** ] , nonsteroidal AR antagonists act as agonists in the presence of  **OSM** .  Ref: 12114440 Clin Cancer Res, 2002
- METHODS IL17 alone, or in combination with IL1,  **IL6** , oncostatin M  [ **OSM** ] , or tumour necrosis factor alpha, was added to bovine nasal cartilage explant cultures.  Ref: 12117676 Ann Rheum Dis, 2002
- To detect potential crosstalk between members of this family, we have extended these observations by assessing the expression levels of other LIF IL 6  [ **IL6** ]  cytokines CNTF,  **OSM** , IL 11, CT 1 and their receptors in the same RC cell model treated with or without LIF or Dex.  Ref: 12110437 Bone, 2002
- Molecular cloning and characterisation of a carp Cyprinus carpio cytokine like cDNA that shares sequence similarity with IL 6  [ **IL6** ]  subfamily cytokines CNTF,  **OSM**  and LIF.  Ref: 12543126 Dev Comp Immunol, 2003
- Oncostatin M  [ **OSM** ]   **OSM**  is an interleukin IL 6  [ **IL6** ]  family cytokine that we have previously shown can synergize with a number of proinflammatory cytokines to promote the release of collagen from cartilage in explant culture.  Ref: 12759253 Am J Pathol, 2003
- Secretion of oncostatin M  [ **OSM** ]  by infiltrating neutrophils regulation of IL 6  [ **IL6** ]  and chemokine expression in human mesothelial cells.  Ref: 12391243 J Immunol, 2002
- Moreover,  **OSM**  synergistically blocked IL 1beta induced CXC chemokine ligand 8 secretion in combination with the IL 6  [ **IL6** ]  sIL 6R complex.  Ref: 12391243 J Immunol, 2002
- Depending on the cell type, FR treatment also enhances the responsiveness to  **OSM**  and IL 6  [ **IL6** ] .  Ref: 12214267 Oncogene, 2002
- Thus, oncostatin M  [ **OSM** ] , an IL 6  [ **IL6** ]  type cytokine, stimulates membrane polarity development in HepG2 cells via the gp130 receptor unit, which activates a protein kinase A dependent and sphingomyelin marked membrane transport pathway from SAC to the apical membrane.  Ref: 12456648 EMBO J, 2002
- IL 6  [ **IL6** ]  and leukaemia inhibitory factor LIF, two  **OSM**  related cytokines, did not significantly modify TF expression at the surface of SMC.  Ref: 12138373 Blood Coagul Fibrinolysis, 2002
- Oncostatin M  [ **OSM** ]   **OSM**  is a member of the IL 6  [ **IL6** ]  LIF or gp130 cytokine family, and its potential role in inflammation is supported by a number of activities identified in vitro.  Ref: 12496442 J Immunol, 2003
- OBJECTIVE The development of megakaryocytes is regulated by thrombopoietin TPO, which binds to the c mpl receptor, and by several other cytokines such as interleukin IL 6  [ **IL6** ] , IL 11, leukemia inhibitory factor LIF, cilliary neurotropic factor CNTF, and oncostatin  **OSM** , which bind to gp130 protein coupled receptors.  Ref: 12135673 Exp Hematol, 2002
- Suboptimal doses of IL17 synergised potently with TNFalpha, IL1,  **OSM** , and  **IL6**  to promote collagen degradation.  Ref: 12117676 Ann Rheum Dis, 2002
- Hepatocyte specific gene expression in BMCs was identified by immunocytochemistry and reverse transcription polymerase chain reaction.RESULTS Bone marrow cell derived hepatocyte like colonies appeared after several days of coculture in medium containing HSLF, oncostatin M  [ **OSM** ]   **OSM**  and hepatocyte growth factor HGF  [ **IL6** ] .  Ref: 12821039 J Hepatol, 2003
- This may be attributable to recruitment of different intermediary signal transduction proteins by  **OSM**  and IL 6  [ **IL6** ] , respectively.  Ref: 12114440 Clin Cancer Res, 2002
- Its activities are shared by IL 6  [ **IL6** ]  related cytokines such as leukemia inhibitory factor and oncostatin M  [ **OSM** ] .  Ref: 12110143 Arthritis Res, 2002
- The IL 6  [ **IL6** ]  family cytokine oncostatin M  [ **OSM** ]  was also capable of activating MAPK, STAT3, STAT1, Akt, and p38 in both WT and SYF cells.  Ref: 12764151 J Biol Chem< MedlineTA>J Biol Chem, 2002
- Continuous treatment with Dex 10 8 mol L inhibited the endogenous production of IL 6  [ **IL6** ] , LIF,  **OSM** , IL 11R, and OSMR, but had no detectable effect on the expression of IL 11, CT 1, CNTF, CNTFR, LIFR, or gp130.  Ref: 12110437 Bone, 2002
- Following the induction of liver injury with acetaminophen APAP, a time dependent increase in liver mRNA expression of IL 6  [ **IL6** ]  and its family members IL 11, leukemia inhibitory factor, and oncostatin M  [ **OSM** ]  was observed in wild type WT mice, suggesting a possible hepatoprotective role played by this cytokine family.  Ref: 12705907 Biochem Biophys Res Commun, 2003
- The IL 6  [ **IL6** ]  type cytokines IL 6  [ **IL6** ] , IL 11, LIF,  **OSM** , CNTF, CT 1 and CLC are an important family of mediators involved in the regulation of the acute phase response to injury and infection.  Ref: 12773095 Biochem J< MedlineTA>Biochem J, 2003
